# ESA-UNet for assisted diagnosis of cardiac magnetic resonance image based on the semantic segmentation of the heart

**DOI:** 10.3389/fcvm.2022.1012450

**Published:** 2022-10-26

**Authors:** Yuanzhe Li, Zhiqiang Liu, Qingquan Lai, Shuting Li, Yifan Guo, Yi Wang, Zhangsheng Dai, Jing Huang

**Affiliations:** ^1^Department of CT/MRI, The Second Affiliated Hospital of Fujian Medical University, Quanzhou, China; ^2^Medical Imaging Department, Guangzhou Twelfth People's Hospital, Guangzhou, China; ^3^Department of Radiology, The First Affiliated Hospital of Zhejiang Chinese Medical University (Zhejiang Provincial Hospital of Traditional Chinese Medicine), Hangzhou, China; ^4^Department of Orthopaedic Surgery, The Second Affiliated Hospital of Fujian Medical University, Quanzhou, China

**Keywords:** cardiovascular disease, artificial intelligence, image segmentation, ESA-UNet, conditional random field

## Abstract

**Background:**

Cardiovascular diseases have become the number one disease affecting human health in today's society. In the diagnosis of cardiac diseases, magnetic resonance image (MRI) technology is the most widely used one. However, in clinical diagnosis, the analysis of MRI relies on manual work, which is laborious and time-consuming, and also easily influenced by the subjective experience of doctors.

**Methods:**

In this article, we propose an artificial intelligence-aided diagnosis system for cardiac MRI with image segmentation as the main component to assist in the diagnosis of cardiovascular diseases. We first performed adequate pre-processing of MRI. The pre-processing steps include the detection of regions of interest of cardiac MRI data, as well as data normalization and data enhancement, and then we input the images after data pre-processing into the deep learning network module of ESA-Unet for the identification of the aorta in order to obtain preliminary segmentation results, and finally, the boundaries of the segmentation results are further optimized using conditional random fields. For ROI detection, we first use standard deviation filters for filtering to find regions in the heart cycle image sequence where pixel intensity varies strongly with time and then use Canny edge detection and Hough transform techniques to find the region of interest containing the heart. The ESA-Unet proposed in this article, moreover, is jointly designed with a self-attentive mechanism and multi-scale jump connection based on convolutional networks.

**Results:**

The experimental dataset used in this article is from the Department of CT/MRI at the Second Affiliated Hospital of Fujian Medical University. Experiments compare other convolution-based methods, such as UNet, FCN, FPN, and PSPNet, and the results show that our model achieves the best results on Acc, Pr, ReCall, DSC, and IoU metrics. After comparative analysis, the experimental results show that the ESA-UNet network segmentation model designed in this article has higher accuracy, intuitiveness, and more application value than traditional image segmentation algorithms.

**Conclusion:**

With the continuous application of nuclear magnetic resonance technology in clinical diagnosis, the method in this article is expected to become a tool that can effectively improve the efficiency of doctors' diagnoses.

## Introduction

Cardiovascular disease has become the number one killer that affects human health in today's society (1). According to domestic statistics, the number of deaths caused by cardiovascular disease has accounted for more than 40% of the total number of deaths from the disease, ranking at the forefront in the composition of deaths, higher than other diseases such as tumors. Research on cardiac diagnosis has always been a research hotspot. With the development of digital imaging technology and the continuous improvement of image segmentation technology (2, 3), medical imaging has become more and more widely used in clinical diagnosis and has become the primary basis for doctors' diagnosis and treatment. Among them, magnetic resonance imaging (MRI) is the most widely used one in the diagnosis of heart disease. Cardiac MRI can provide clearer information on cardiac structure, myocardial motion, and histological features.

Although MRI technology plays an important role in the diagnosis of heart disease, medical imaging itself is complex and requires extremely high accuracy of results. At present, the analysis of medical images is mainly completed by experienced doctors. Since automatic segmentation and diagnosis cannot meet clinical needs, they can only be used as auxiliary supplements. However, the workload required for manual analysis by radiologists is large and time-consuming and is affected by the subjective experience, environment, and working status of different radiologists, and the results vary from person to person. The sketched results are not 100% reproducible. In recent years, with the rapid development of artificial intelligence and deep learning, the use of computer-aided diagnosis and treatment can significantly improve the efficiency of diagnosis. Classification and semantic segmentation are commonly used, in which semantic segmentation can not only diagnose the type of disease but also point out the location of the disease, which is an effective auxiliary means for intelligent diagnosis. Computer technology can be harnessed to locate and segment the region of interest (ROI) in the medical image, identify the pixel points in the ROI area, and obtain the characteristic parameters of the ROI, to provide reliable reference information for the subsequent analysis of the disease and evaluation of treatment, and assist doctors to carry out diagnosis and treatment. Medical image segmentation is a key step in medical image processing and is crucial for the next step of diagnosis and treatment (4).

At present, there are many segmentation methods widely used at home and abroad. The traditional segmentation methods include edge-based image segmentation, region-based image segmentation, and image segmentation combined with specific theories, etc. (5–8). Zhang et al. (9) proposed a medical image clustering and segmentation algorithm, which uses a dictionary as the clustering center of clustering segmentation, and determines the cluster attribution through sparse representation to achieve medical image segmentation.

In recent years, deep learning algorithms have shown powerful capabilities in image processing, especially the convolutional network model for medical image segmentation is better than traditional segmentation algorithms. M.R. Avendi et al. (10) used a convolutional neural network to locate the left ventricular region of the heart from cardiac MRI, and then used a stack auto-encoding algorithm model to outline the initial shape of the left ventricle. Long et al. (11) proposed a fully convolutional neural network segmentation method, which uses transposed convolution to restore the feature map to the original image size to achieve pixel-level segmentation, and then realize the entire image segmentation. Nasresfahani et al. (12) extracted the ROI region in the image processing stage and used a fully convolutional neural network to segment the left ventricle.

Although convolutional networks have achieved promising results in MRI segmentation tasks, they lack efficiency in capturing global contextual information due to the inherent limitations of convolutions. This results in large differences in texture, shape, and size of segmented hearts from patient to patient. For two pixels that are far apart, many layers of convolution are often needed to achieve, but too deep can easily affect the training effect.

For this reason, the self-attention mechanism based on CNN features is proposed to solve this problem (13, 14). The attention mechanism was first proposed by Vaswani et al. (15) to solve the problem of machine translation. The attention mechanism can adjust the learned weights to make important features more weighted. Wang et al. (16) introduced the attention mechanism into computer vision for the first time and adjusted the weights of feature maps by calculating the correlation between pixels. Subsequently, attention mechanisms have been widely used in the field of medical images. Li et al. (17) designed an attention-based nested UNet model to segment liver tumor images. The network proposes an attention gate module, which can aggregate the encoder and upsampled information while adjusting the weights. Fan et al. (18) proposed a network Inf-Net for segmenting CT images of COVID-19. The network utilizes a set of implicit reverse attention modules and explicit edge attention guidance to establish the relationship between regions and boundaries. Liu et al. (19) designed a CANet network based on an attention conditional random field to segment gliomas, where attention can regulate the amount of information flowing between different features. Dou et al. (20) designed a segmentation network with deep attention module convolution kernels to segment fetal cortical plates.

The attention mechanism can obtain long-range feature information and adjust the weight of feature points by aggregating the correlation information of global feature points. Although the attention mechanism has significantly improved the recognition accuracy of the model, attention mechanism has the problems of high time complexity, slow training speed, and many weight parameters. To ensure rich semantic information, the semantic segmentation network usually uses large-sized feature maps, which causes the time complexity of the model to be too high. To solve the problem of time complexity brought by the attention mechanism, tensor decomposition can well reduce the time complexity of the attention mechanism. Tensor decomposition is widely used in computer vision acceleration. According to tensor decomposition theory (21), high-rank tensors can be decomposed into linear combinations of low-rank tensors. Lebedev et al. (22) proposed a method for accelerating convolutional layers in large convolutional networks based on CP tensor decomposition. The method first decomposes a high-rank tensor of four-dimensional convolution kernels into multiple rank tensors and then uses a rank-one convolution kernel to speed up network training. Wu et al. (23) decomposed the weight matrix of the fully connected layer into a Kronecker product of multiple sub-tensors to approximate the fully connected layer while reducing the parameters in the neural network. Sun et al. (24) designed a tensor decomposition method for network optimization. This method realizes the compression of the model by using the characteristic that the weight tensors between each layer of the network contain the same or independent components, and decomposes the sequence of the coupling tensors on the shared network structure.

Chen et al. (25) proposed RecoNet, a three-dimensional contextual feature representation semantic segmentation model. The model achieves the approximation of a high-rank tensor by the linear combination of low-rank sub-tensor features, which significantly reduces the computational complexity of the model compared to the original feature map. The above methods usually replace a high-rank tensor with multiple low-rank tensors. Tensor decomposition can decompose the original tensor with high computational complexity into a set of low-rank sub-tensors. By calculating the low-rank sub-tensor, the parameter quantity of the network model can be reduced and the network can be accelerated at the same time. Although the tensor decomposition method can improve the compression rate of the model, the recognition efficiency of the model will decrease when the model compression rate is high. To alleviate the problem of low recognition efficiency caused by tensor decomposition, this article uses a shared structure in the network to improve the performance of the model.

Based on the above analysis, this article proposes a deep learning-based cardiac MRI segmentation scheme. We first performed pre-processing of the unsegmented MRI, including the region of interest detection (ROI), data normalization, and data enhancement of the cardiac MRI data. ROI detection is based on Canny edge detection, and ROI detection is performed by using the Hough transform for the detection of circles to narrow down the segmentation region. We then artificially augment the experimental dataset using multiple data augmentation means. For the semantic segmentation network, we introduce a self-attention module in the traditional convolutional network structure. We propose ESA-UNet, a U-shaped semantic segmentation network, which is embedded with a low-rank tensor self-attention structure. ESA-UNet uses an encoding-decoding structure to realize the fusion of feature information of different scales. To obtain richer semantic information and reduce the complexity of the self-attention model, this article designs a low-rank tensor self-attention reconstruction module, decomposes high-rank tensors into low-rank tensors, and uses low-rank tensors to construct the Self-attention feature maps, and then aggregate multiple low-rank self-attention maps to generate high-rank self-attention feature maps. For the network segmentation results, we performed a further optimization of tumor boundaries using conditional random fields. We conduct a full experimental analysis of the ACDC dataset, and the results show that our proposed segmentation method outperforms other methods. This method will play an extremely important role in the diagnosis, treatment, and prognosis of heart disease.

## Method

### Datasets

In this article, the Department of CT/MRI at the Second Affiliated Hospital of Fujian Medical University collects cardiac MRIs from 150 different patients. The dataset includes 100 training samples and 50 testing samples. Each training sample contains expert manual segmentation and annotation results of the right atrium, left atrium, and aorta at end-diastole (ED) and end-systole (ES). The MRI data for each patient consisted of 28 to 40 frames of a series of short-axis image slices of the entire cardiac cycle from the bottom to the top of the left ventricle. The spatial resolution of each slice averages 235–263 voxels.

### Pre-processing

To ensure the segmentation effect of the segmentation network, we first perform sufficient pre-processing on the MRI. The pre-processing steps include region of interest detection (ROI), data normalization, and data enhancement for cardiac MRI data.

Region of interest detection is divided into two steps: filtering and edge detection. The filtering operation uses a standard deviation filter to find regions of the cardiac cycle image sequence where the pixel intensity varies strongly over time. Edge detection uses Canny edge detection (26) and the Hough transform technique (27) to find the region of interest containing the heart.

The Canny edge detection consists of the following four steps.

#### Remove noise in the image *via* a gaussian smoothing filter

In the process of image edge detection, the edge and noise of the image are difficult to distinguish, and the edge detection algorithm alone cannot eliminate the influence of noise on the edge detection process and results, so the original image needs to be preprocessed. Common filtering methods in image pre-processing include mean filtering, median filtering, and Gaussian filtering. Compared with mean filtering and median filtering, Gaussian filtering can well preserve the grayscale distribution in the image when smoothing the image.

#### Calculate image gradient strength and orientation

The basic idea of Canny's algorithm is to find the position of the strongest gray intensity change in an image, that is, the gradient direction. The gradient of each pixel in the smoothed image is calculated by the Sobel operator. First, the following convolution arrays *S*_*x*_ and *S*_*y*_ are used to obtain the gradients *G*_*x*_ and *G*_*y*_ of the original image A along the horizontal (x) and vertical (y) directions, respectively:


(1) $$\begin{array}{l} G_{x}=S_{x}*A=\left[\begin{array}{ccc} -1 & 0 & +1\\ -2 & 0 & +2\\ -1 & 0 & +1 \end{array}\right]*A \end{array}$$

(2) $$\begin{array}{l} G_{y}=S_{y}*A=\left[\begin{array}{ccc} -1 & -2 & -1\\ 0 & 0 & 0\\ +1 & +2 & +1 \end{array}\right]*A \end{array}$$

Then use the following equation to find the gradient magnitude of each pixel:


(3) $$\begin{array}{l} G=\sqrt{G_{x}^{2}+G_{y}^{2}} \end{array}$$

A large gradient metric value G will be obtained in places with drastic changes (at the boundary), but these boundaries are usually very thick, and it is difficult to demarcate the real position of the boundary. To demarcate the boundary, the direction information of the gradient is also required:


(4) $$\begin{array}{l} \theta =\text{arctan}\left(\frac{G_{x}}{G_{y}}\right) \end{array}$$

#### A non-maximum suppression technique is applied to eliminate edge false detections

Each pixel's gradient direction was set to one of the following values: (0°, 45°, 90°, 135°, 180°, 225°, 270°, and 315°). We judge whether it is an edge by comparing the gradient strength of the pixel and the two pixels in the positive and negative gradient directions. If the gradient strength of the pixel is the largest, it will be retained, and it will be regarded as an edge. This way we will get one of the brightest thin lines at the border, and the edges of the image will be noticeably thinner.

#### A double threshold is applied to decide possible edges

The technique of double threshold is applied in the Canny algorithm, that is, an upper threshold T1 and a lower threshold T2 are set. If the gradient value of the pixel point exceeds T1, it is called a strong edge, and the one in between is called a weak edge, otherwise, it is not an edge. The larger the T1, the more severe the gradient change in strong edge pixels. Canny recommends setting T1:T2 to 2:1.

### Segmentation network model

**Figure 1:**
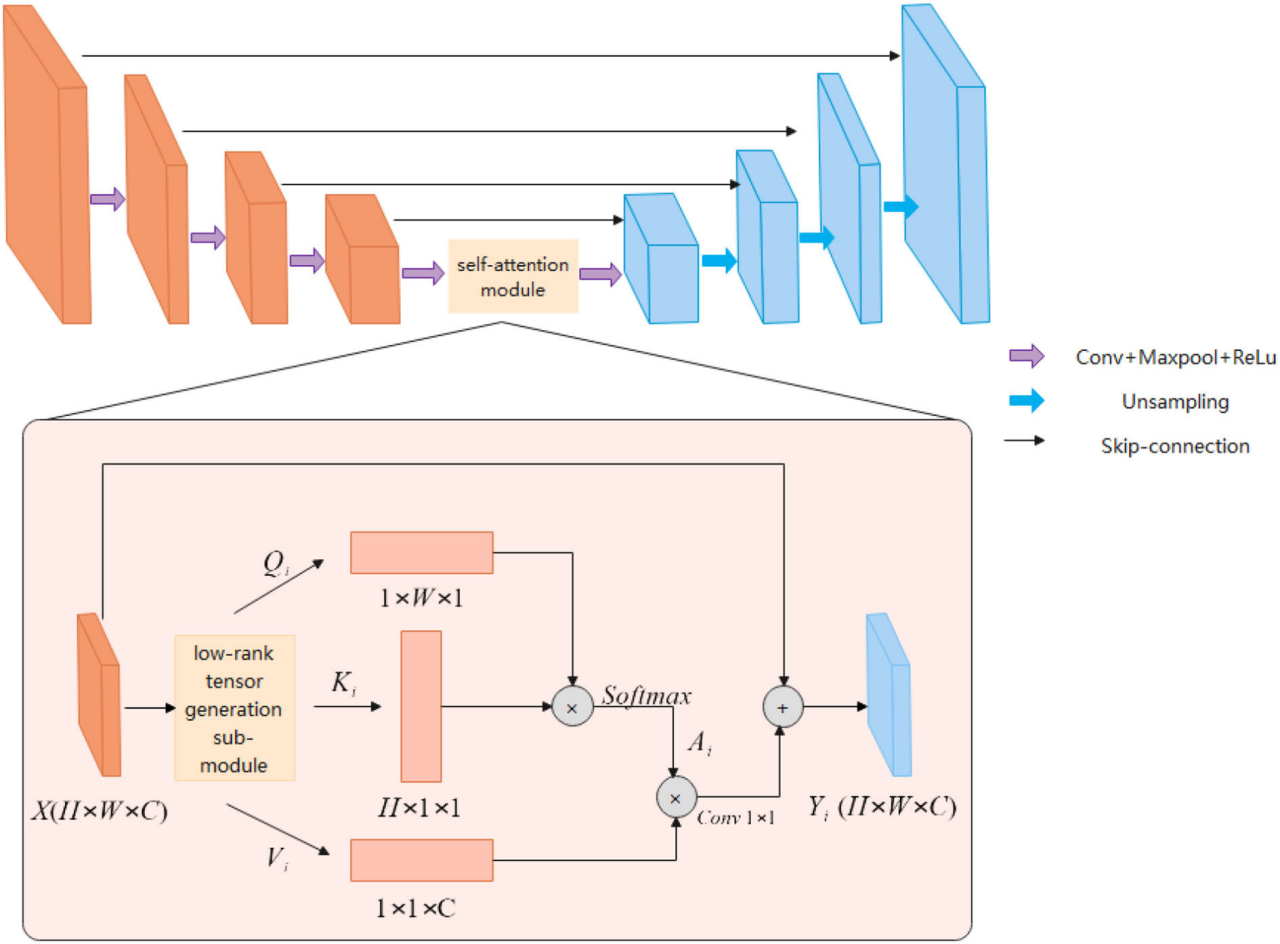
ESA-UNet network structure diagram.

The ESA-Unet proposed in this article is shown in Figure 1. The network mainly consists of three parts: encoder, decoder, and low-rank self-attention reconstruction module. ESA-UNet jointly designs a self-attention mechanism and multi-scale skip connections based on a convolutional network, which effectively makes up for the problem that convolution is difficult to model long sequences. This ensures that the global context in cardiac MRI is not completely ignored, effectively enhancing the functionality and robustness of the traditional U-shaped architecture. ES-UNet network consists of three parts: decoder, encoder, and self-attention module.

#### Encoder

The encoder is a structure used to extract image features. The encoder uses a five-layer residual connected downsampling layer to obtain multi-scale feature information of five different levels of the image. The low-level features are mainly used to obtain the detailed features and position information of the image, and the high-level features are abstract semantic features information. Each downsampling layer consists of two consecutive 3 × 3 convolutional layers, a RELU activation function, and a 2 × 2 max-pooling layer. And the downsampling is connected in a residual structure. The residual structure can obtain richer semantic information by extending the depth of the network.

#### Decoder

The main function of the decoder is to gather feature information at different levels. The decoder first uses a cascaded upsampling layer to restore the image resolution to the original size of *H*×*W*, and finally uses a 1 × 1 convolutional layer. Decrease the number of channels to get the final segmentation map. Each of these upsampling layers consists of a 2 × 2 Up-Conv, a 3 × 3 convolutional layer, and a RELU layer. We still maintain the U-shaped structure of UNet and concatenate the features extracted in the encoder with the upsampled feature map to fuse the feature information of different levels. This can effectively avoid the loss of low-level information, such as organ shape and boundary.

#### Low-rank tensor self-attention module

The attention module is used to obtain richer contextual information. Although the convolutional structure can expand the receptive field and extract rich information by stacking more layers, the deeper convolutional layer structure is not good for global information to obtain. The attention module can adjust the global information, and each point in the image will calculate the correlation with other points. The correlation information obtained through the attention feature map adjusts the pixel weights in the picture, the weights belonging to the same other points will be aggregated, and the pixel point information of different categories will be suppressed to highlight the important parts of the picture. The attention mechanism can obtain rich semantic information, but the amount of computation will be relatively large. The low-rank tensor self-attention reconstruction module LRSAR Block proposed in this article can well solve the computationally complex problem.

The low-rank tensor self-attention module includes three parts: low-rank tensor generation sub-module, low-rank self-attention sub-module, and high-rank tensor reconstruction sub-module.

##### Low-rank tensor generation sub-module

The low-rank tensor generation sub-module can perform high-rank tensor decomposition along the width, height, and channel dimensions. According to the CP tensor decomposition theory, a high-rank tensor can be decomposed into a linear combination of multiple-rank tensors. A rank tensor can be composed of the outer product of three one-dimensional vectors. According to the definition of a rank tensor, the author decomposes the high-rank tensor along the width, height, and channel dimensions to generate multiple one-dimensional vectors. These one-dimensional vectors are input into the low-rank tensor self-attention sub-module to generate a rank tensor. The high-rank tensor extracted by the coding layer is input into three low-rank tensor generation modules to extract the low-rank tensor feature information. The high-rank tensor is input to the low-rank tensor generation module multiple times to generate multiple different low-rank tensor features. That is, the high-rank tensor feature *X* is input to the low-rank tensor generation sub-module *s* times, which will generate *s* different low-rank tensor features. The low-rank tensor decomposed along the same dimension has the same network structure, but different parameter information.

The feature map *X* will be input to the low-rank tensor generation sub-module multiple times along the three dimensions of height, width, and channel to generate different feature vectors (**Q**_1_, **K**_1_, **V**_1_), (**Q**_2_, **K**_2_, **V**_2_)…(**Q**_*i*_, **K**_*i*_, **V**_*i*_), (**Q**_*s*_, **K**_*s*_, **V**_*s*_).**Q**_*i*_, **K**_*i*_ and **V**_*i*_ represent the one-dimensional vectors generated by decomposition along with the height, width, and channel dimensions, respectively, and represents the number of one-dimensional vectors generated along a certain dimension. Equations (6–8) Represent *Q*_*i*_, *K*_*i*_ and *V*_*i*_, respectively. These low-rank feature vectors are passed through the low-rank self-attention module to generate different low-rank self-attention sub-feature maps**Y**_1_, **Y**_2_, …, **Y**_*i*_, …, **Y**_*s*_. Each low-rank tensor generation sub-module consists of global average pooling (GAP), fully connected layer (FC), and sigmoid activation function, and generates a one-dimensional feature vector for self-attention feature maps. The principle of global average pooling is to first slice the high-rank tensor along a certain dimension and perform global average pooling for each slice matrix. Through global average pooling, each element in each vector aggregates the corresponding slice matrix information. The fully connected layer can realize the aggregation of all element information by any element in the vector. The sigmoid activation function can enhance the nonlinear fitting ability of the network, and map the feature information to the range of 0–1, highlighting the important feature information in the feature vector. The low-rank tensor generation module in the literature (28) uses the convolution structure, and this article replaces the convolution with the FC layer. Each feature point in the FC layer will be aggregated with other feature information, while the single-layer convolutional structure can only aggregate local feature information. The FC layer parameter information of different low-rank tensor features is different. Although the FC layer will increase the number of parameters, the feature dimension of the last layer of the encoding layer is relatively low, and the number of parameters will not increase much.


(5) $${\text{Q}}_{i}=\left(q_{1},q_{2},\cdots ,q_{m},\cdots ,q_{H}\right),i=1,2,\cdots ,s;m=1,2,\cdots ,H\;$$

(6) $${\text{K}}_{i}=\left(k_{1},k_{2},\cdots ,k_{n},\cdots ,k_{W}\right),i=1,2,\cdots ,s;n=1,2,\cdots ,W\;$$

(7) $${\text{V}}_{\text{i}}=\left(v_{1},v_{2},\cdots ,v_{l},\cdots ,v_{C}\right),i=1,2,\cdots ,s;l=1,2,\cdots ,C$$

##### Low-rank self-attention sub-module

**Figure 2:**
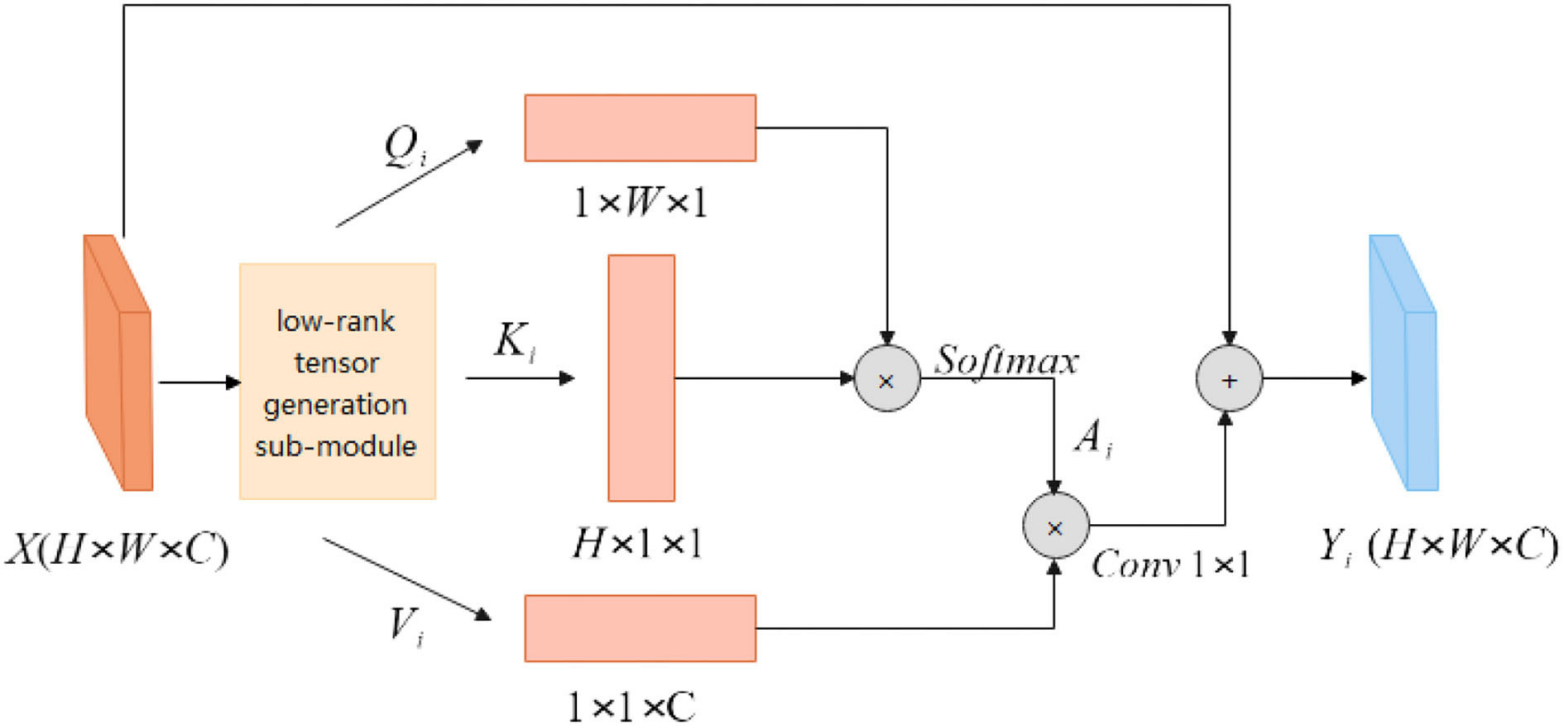
Low-rank self-attention sub-module.

As shown in Figure 2, the feature map **X**∈**R**^*H*×*W*×*C*^ is first input into the low-rank tensor generation sub-module to generate multiple different low-rank tensors **Q**_*i*_, **K**_*i*_ and **V**_*i*_. The height feature **Q**_*i*_ is multiplied by the width feature **K**_*i*_ to obtain the spatial similarity matrix $A_{i}\in R^{H\times W\times 1}$, which is activated by the Softmax layer. The specific calculation process is shown in equation (9). Equation (10) is a more detailed explanation of the feature similarity matrix **A**_*i*_, *a*_*mn*_ represents each point on the spatial similarity matrix, *q*_*m*_ and *k*_*n*_, respectively, represent the width feature information, and height feature information. The obtained spatial attention feature map **A**_*i*_ has no correlation information between channels, and feature **V**_*i*_ aggregates the information between channels. The attention feature map **A**_*i*_ is multiplied by the channel attention information **V**_*i*_ to obtain three-dimensional attention information. The calculation process is shown in Equation (11). The input feature map *X* is added to the attention feature to obtain long-range semantic information features and the feature map **Y**_*i*_ is obtained.


(8) $${\text{A}}_{\text{i}}\text{=softmax}\left({\text{Q}}_{\text{i}}{\text{×K}}_{\text{i}}\right)$$

(9) $$a_{mn}=\frac{\exp(q_{m}k_{n})}{\sum_{m=1}^{W}\exp(q_{m}k_{n})}$$

(10) $${\text{Y}}_{\text{i}}\text{=X+Conv1 × 1}\left({\text{A}}_{\text{i}}{\text{×V}}_{\text{i}}\right)$$

The self-attention module non-local block calculates the correlation between any two points in the image when calculating the similarity of pixels. For the feature map *X*, the time complexity of the self-attention module is *O*(*H*×*W*×*H*×*W*), while the LRSAR block only needs to calculate the outer product of two vectors, and the time complexity is *O*(*H*×*W*), lower degree. Compared with the self-attention module, the LRSAR-Net proposed in this article has lower time complexity and faster speed.

##### High-rank tensor reconstruction sub-module

According to tensor decomposition theory, high-order tensors can be decomposed into linear combinations of multiple rank-one tensors. The feature map *X* passes through the low-rank self-attention module to generate multiple rank-one attention feature maps **Y**_*i*_, and **Y**_*i*_ only contains low-level semantic information. The rank-one attention feature map **Y**_*i*_ is generated by different parameters low-rank tensor generation modules, so the feature information contained in different rank-one attention feature maps is different. The authors introduce a learnable weight parameter λ_*i*_ before each rank-one attention feature map **Y**_*i*_, which is adjusted with training. Each low-rank self-attention feature map is multiplied by the corresponding weight parameter λ_*i*_, and then combined into a high-rank self-attention tensor *Y*. The tensor reconstruction method is shown in Equation(12). The high-rank attention feature map *Y* contains rich semantic information, realizes the aggregation of global feature information, and reduces the computational cost of self-attention feature maps. In this experiment, to balance the complexity of the model and the amount of computation, *s* is set to 4.


(11) $$Y=\sum {}_{i}^{n}2mu\lambda_{i}Y_{i}$$

### Conditional random fields optimize segmentation boundaries

After network segmentation, we use a conditional random field (CRF) to further optimize the segmentation boundary.

For the probability map *U* after the output of the neural network, we can use the following equation to describe the predicted value of each pixel. *X* = {*x*_1_, *x*_2_, …, *x*_*n*_} represents each pixel feature point on the probability map, *Y* = {*y*_1_, *y*_2_, *y*_3_, …, *y*_*n*_} represents each point according to its texture, gray value, and other attributes and surrounding The label for the probability prediction of the point.


(12) $$\begin{array}{l} P\left(y|x\right)=\frac{1}{Z\left(x\right)}exp(\sum {}_{i\in U}^{}\sum {}_{j\in U\left(x_{i}\right)}^{}T_{i,j}\left(y_{j},y_{i},x_{i},i\right)\\ \;+\sum {}_{i\in U}^{}S_{i}\left(y_{i},x_{i}\right)) \end{array}$$

Among them, *U*(*x*_*i*_) represents the points around *x*_*i*_, ,*T*_*i, j*_ is the function of the feature transfer between the i-th point and the surrounding points, *S*_*i*_ is the state feature function about the i-th point, and *Z*(*x*) is the normalization function:


(13) $$\begin{array}{l} Z\left(x\right)=\sum_{y\epsilon Y}P\left(y|x\right) \end{array}$$

### Evaluation metrics for segmentation results

The evaluation indicators for segmentation results in semantic segmentation problems include accuracy (Acc), precision (Pre), recall (Re), F1 score (F1), intersection of union (IoU), and dice similarity coefficient (DSC) (29, 30). Calculating these evaluation metrics requires the use of four commonly used metrics for prediction results, namely the true positive (TP), false positive (FP), true negative (TN), and false negative (FN). The true negative mainly refers to the situation that the model predicts that the pixels belong to the background area and are consistent with the actual gold standard. False negatives mainly refer to the situation that the model predicts that the pixels belong to the background area, but are opposite to the actual gold standard.


$$\begin{array}{l} Acc=\frac{TP+TN}{TP+TN+FP+FN} \end{array}$$

(14) $$\begin{array}{l} Pre=\frac{TP}{TP+FP} \end{array}$$

$$\begin{array}{l} Re=\frac{TP}{TP+FN} \end{array}$$

$$\begin{array}{l} F1=\frac{2^{*}Pre^{*}Re}{Pre+Re}\\ IOU=\frac{I_{1}\cap I_{2}}{I_{1}\cup I_{2}}\\ DSC=\frac{2^{*}|I_{1}\cap I_{2}|}{\left|I_{1}|+|I_{2}\right|} \end{array}$$

## Results and discussion

### ROI detection results

**Figure 3:**
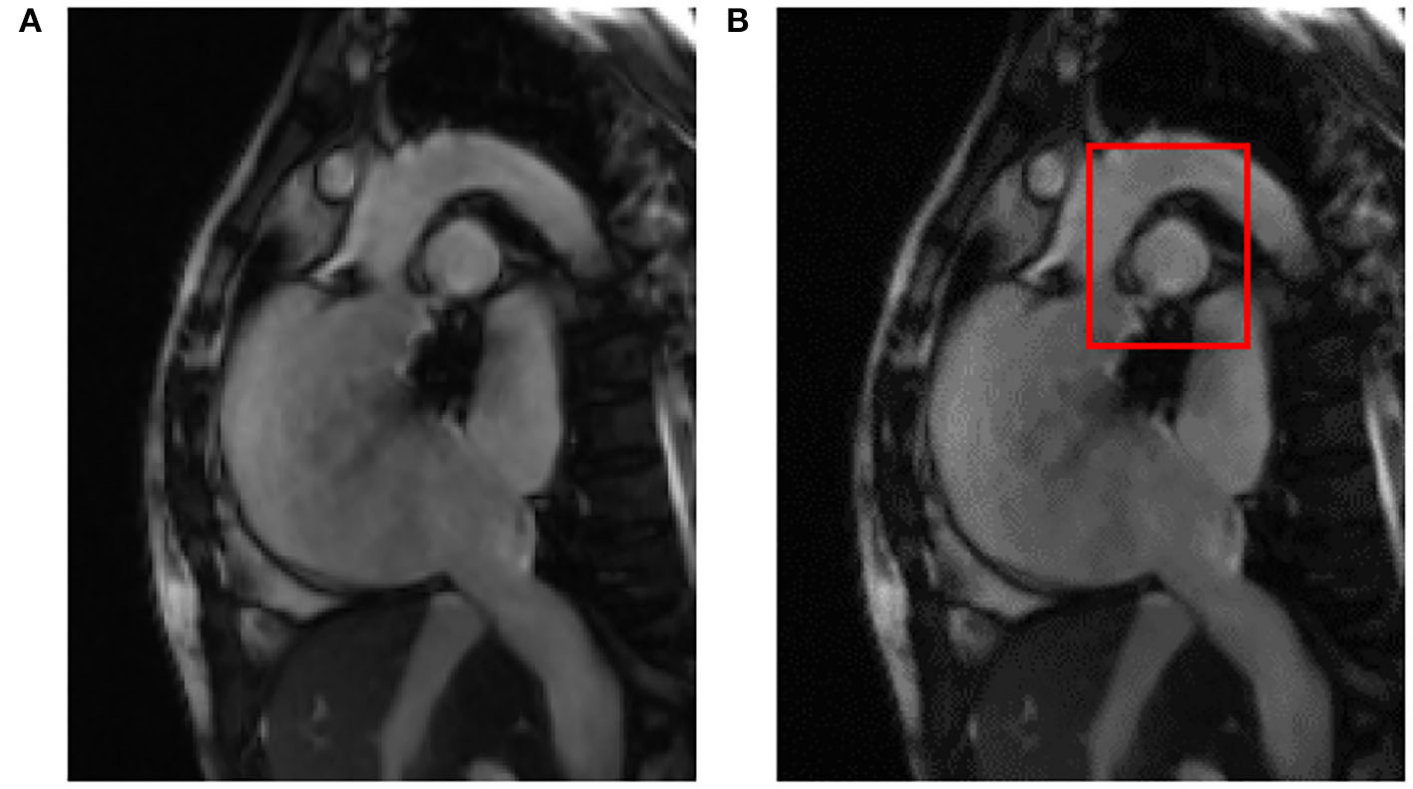
ROI detection results based on **(A)** the original image and **(B)** the detected target area.

After reading the original cardiac MRI data, after the pre-processing step including ROI detection, the results are shown in Figure 3, where Figure 3A is the input of the original cardiac MRI data and Figure 3B is the aorta as the center after ROI detection A gray mask of the ROI containing the aorta is drawn.

**Figure 4:**
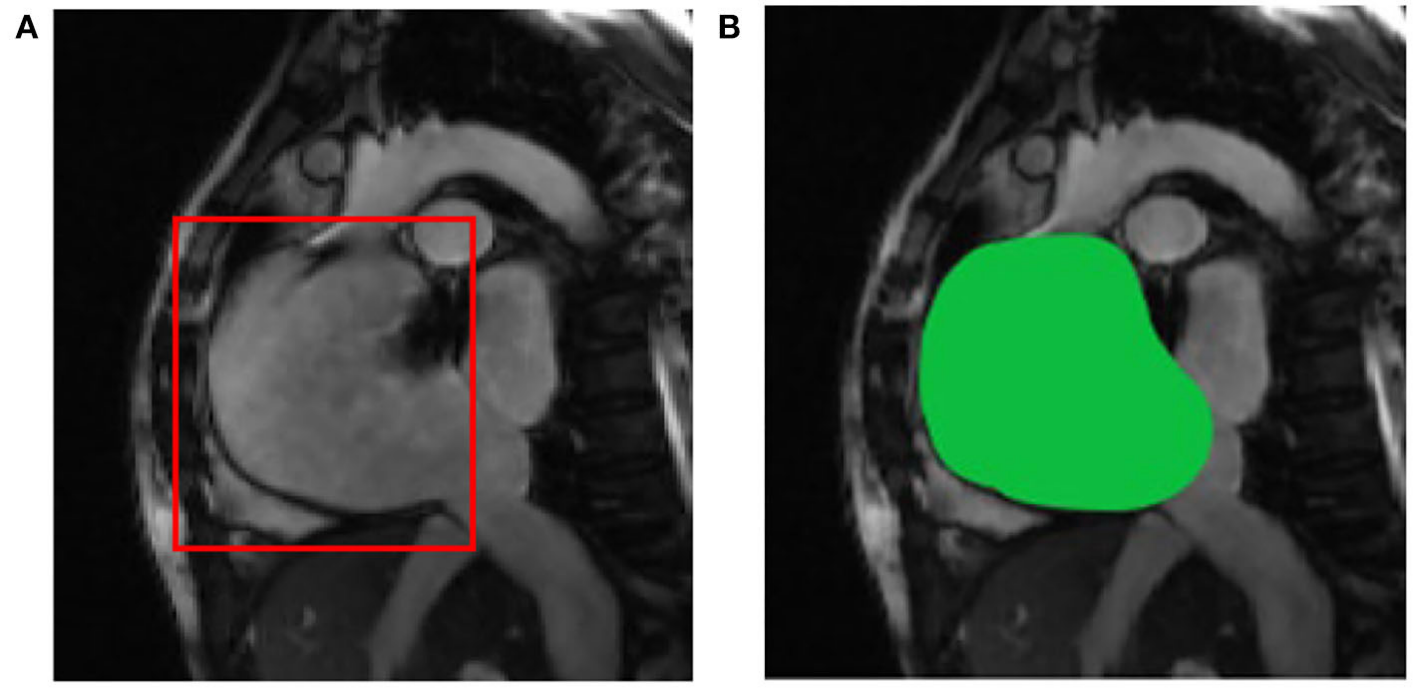
ROI detection and segmentation results based on **(A)** the original two-chamber image; and **(B)** the right atrium image after ROI detection.

After image data standardization and data enhancement processing, according to the ROI area center and area radius obtained by ROI detection, the image data are cropped into a 128 × 128 block with the ROI center, i.e., the aorta as the center, as the input of the deep learning segmentation network. Compared with the raw cardiac MRI slice data with an average spatial resolution of 235–263 voxels per slice as input directly, the GPU memory size occupied by the same model training is reduced from more than 10 GB to less than 6GB. The results of ROI detection and segmentation are shown in Figure 4. In Figure 4A is the input image and Figure 4B is the 128 × 128 image after ROI.

### Image segmentation results

**Figure 5:**
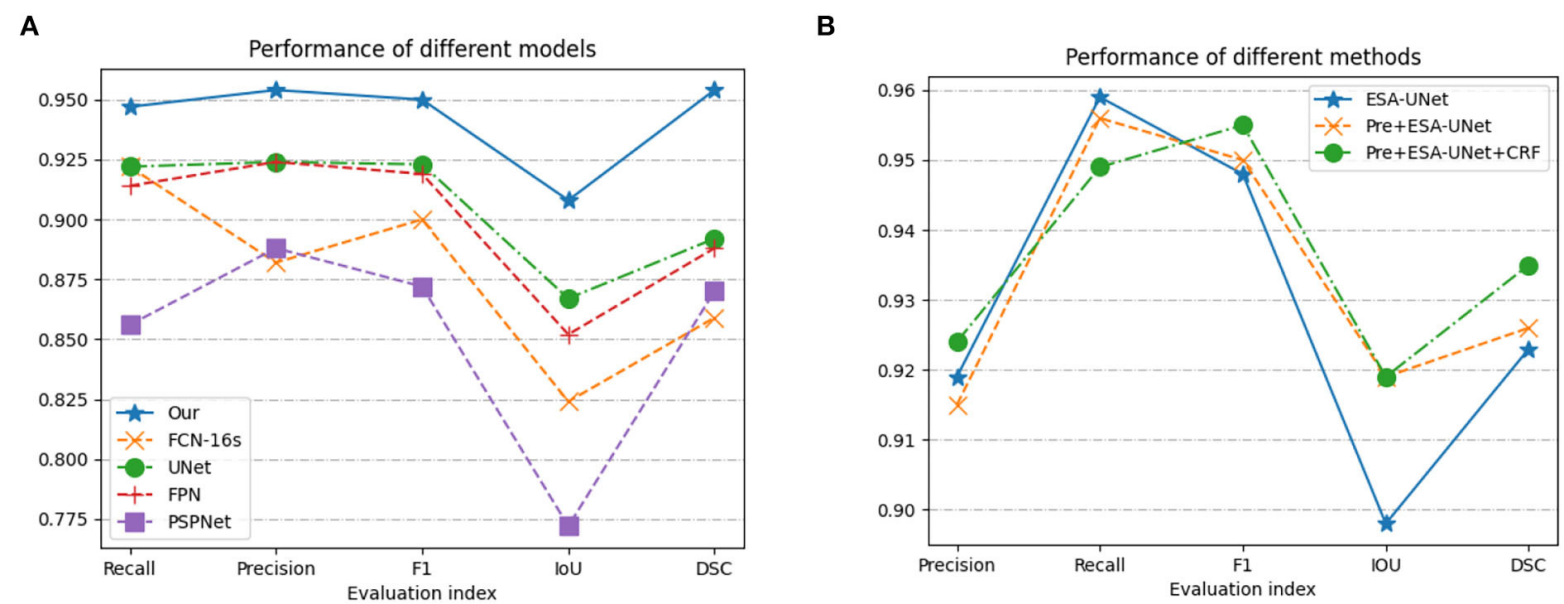
Performance based on **(A)** model methods on different indicators; and **(B)** three methods combination proposed.

We have fully experimented with our method with a variety of excellent convolution-based methods (31–36). From the data in Table 1, we can see that our model performs well in all indicators. On the important indicators Iou and DSC, our model achieves 0.899 and 0.915, respectively. It can also be seen from Figure 5 that pre-processing can improve the IoU indicator to 0.898. Conditional random fields can improve the IoU indicator to 0.915. As can be seen from the data, both pre-processing and conditional random fields can facilitate segmentation.

**Table 1:** Results of different method models on the cardiac MRI.

**Model**	**Pr**	**Re**	**F1**	**IOU**	**DSC**
FCN-16s	0.902	0.862	0.880	0.804	0.839
FCN-8s	0.921	0.853	0.881	0.820	0.856
PSPNet	0.836	0.868	0.852	0.752	0.850
MSFCN	0.861	0.916	0.886	0.821	0.854
MSRN	0.873	0.925	0.898	0.833	0.867
FPN	0.904	0.904	0.909	0.832	0.868
UNet	0.902	0.904	0.903	0.857	0.872
Our(ESA-Unet)	0.919	0.939	0.928	0.888	0.903
Our(ESA-Unet + preprocessing)	0.925	0.936	0.930	0.898	0.906
Our(ESA-Unet + preprocessing + CRF)	0.944	0.929	0.935	0.899	0.915

**Figure 6:**
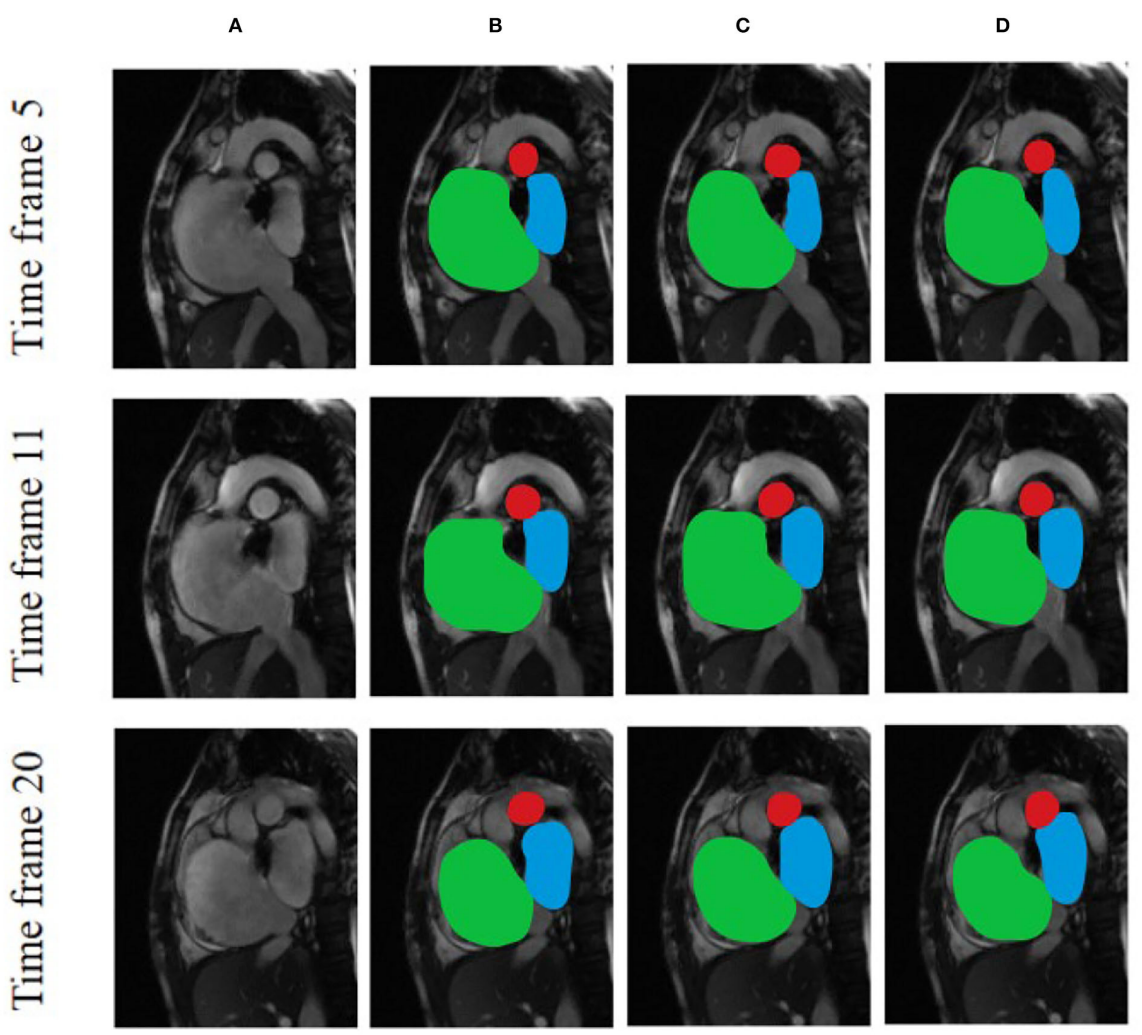
Comparison of the effects of different segmentation models using various cardiac MRI slices based on **(A)** Original and unprocessed images; **(B)** ESA-UNet; **(C)** UNET; and **(D)** FCN.

**Figure 7:**
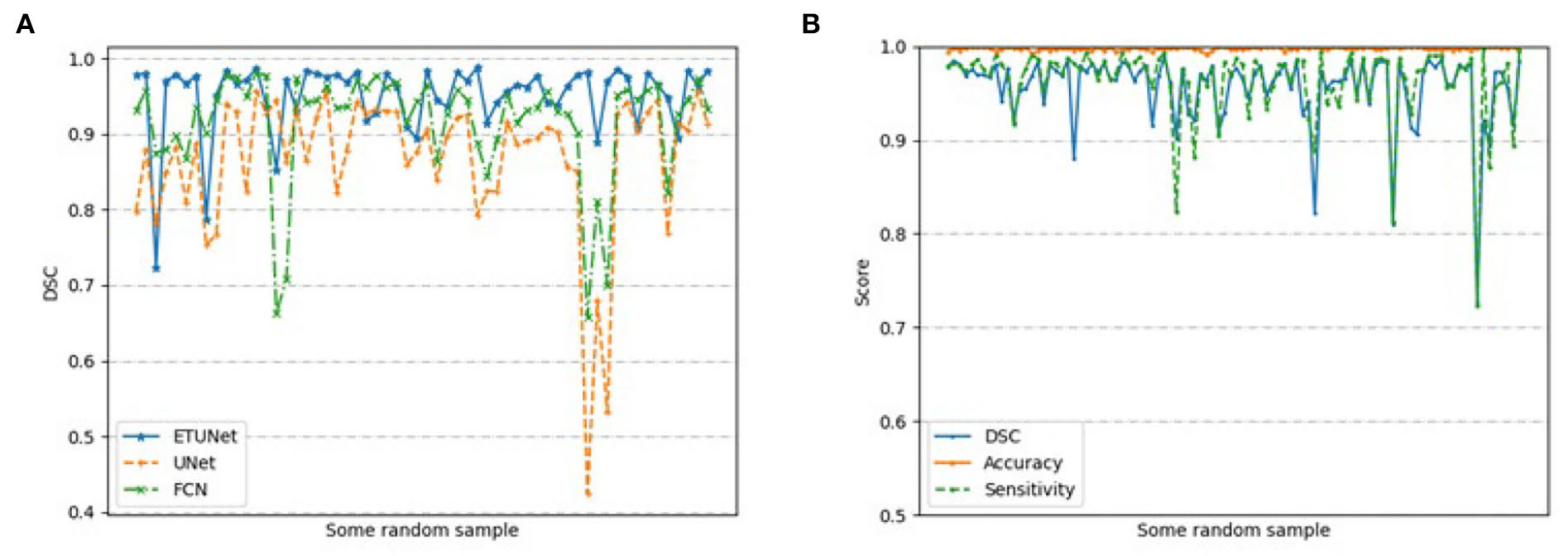
Performance based on **(A)** different models on randomly selected samples; and **(B)** our segmentation model on 200 randomly selected samples.

Figure 6 compares the segmentation effects of different segmentation models on cardiac MRI. Figure 7 shows the performance of different models on different metrics on some specific datasets. As can be seen from the segmentation effect in Figure 6, the segmentation effect of ESA-UNet is better than that of other pure convolutions, and the segmentation results are more robust. Especially when segmenting small target areas such as the red part, other models perform very unstable, but our model can still segment accurately. Figure 7 shows the performance comparison between different models on some characteristic data samples. We can find that our method is more robust in terms of various indicators, and it is not easy to produce samples with poor segmentation results. However, the segmentation results of other methods are more volatile and prone to poorly segmented samples (37, 38).

## Conclusion

In this article, we propose a set of solutions for assisting cardiac MRI diagnosis based on semantic segmentation technology. We first preprocessed the input MRI data, we first filtered using a standard deviation filter to find regions of the cardiac cycle image sequence where pixel intensity varied strongly with time and then used Canny edge detection and Hough transform techniques to find regions containing the heart area of interest. Then, the image is input into the ESA-Unet model network, and the preliminary segmentation results are obtained through the encoder, self-attention module, and decoder; finally, we use the conditional random field to reprocess the segmented image to optimize its segmentation boundary. The results show that our method has a good segmentation effect, which facilitates the diagnosis of clinical cardiovascular diseases and improves the efficiency and accuracy of diagnosis.

In future, we will continue to improve the experiment in combination with clinical practice and try to introduce implicit feature information such as texture to optimize the error of complex segmentation boundaries and further improve the segmentation accuracy.

## Data availability statement

The original contributions presented in the study are included in the article/supplementary material, further inquiries can be directed to the corresponding author/s.

## Ethics statement

The studies involving human participants were reviewed and approved by the Second Affiliated Hospital of Fujian Medical University. The patients/participants provided their written informed consent to participate in this study.

## Author contributions

YW, QL, SL, and ZL were responsible for the integrity of the data analysis. YL oversaw manuscript drafting and study design. YW and JH oversaw data interpretation. All authors contributed to the article and approved the submitted version.

## Funding

This work was sponsored by Fujian Provincial Health Technology Project (2020CXA045 and 2021QNA038).

## Conflict of interest

The authors declare that the research was conducted in the absence of any commercial or financial relationships that could be construed as a potential conflict of interest.

## Publisher's note

All claims expressed in this article are solely those of the authors and do not necessarily represent those of their affiliated organizations, or those of the publisher, the editors and the reviewers. Any product that may be evaluated in this article, or claim that may be made by its manufacturer, is not guaranteed or endorsed by the publisher.
